# Reduction in patient burdens with graphical computerized adaptive testing on the ADL scale: tool development and simulation

**DOI:** 10.1186/1477-7525-7-39

**Published:** 2009-05-05

**Authors:** Tsair-Wei Chien, Hing-Man Wu, Weng-Chung Wang, Roberto Vasquez Castillo, Willy Chou

**Affiliations:** 1 Department of Rehabilitation, Chi-Mei Medical Center, Taiwan, ROC; 2 Department of Hospital and Health Care Administration, Chia-Nan University of Pharmacy and Science, Tainan, Taiwan, ROC; 3 Department of Educational Psychology, Counseling and Learning Needs, Hong Kong Institute of Education, Hong Kong; 4 Director SILAIS, Carazo, Nicaragua, Central America

## Abstract

**Background:**

The aim of this study was to verify the effectiveness and efficacy of saving time and reducing burden for patients, nurses, and even occupational therapists through computer adaptive testing (CAT).

**Methods:**

Based on an item bank of the Barthel Index (BI) and the Frenchay Activities Index (FAI) for assessing comprehensive activities of daily living (ADL) function in stroke patients, we developed a visual basic application (VBA)-Excel CAT module, and (1) investigated whether the averaged test length via CAT is shorter than that of the traditional all-item-answered non-adaptive testing (NAT) approach through simulation, (2) illustrated the CAT multimedia on a tablet PC showing data collection and response errors of ADL clinical functional measures in stroke patients, and (3) demonstrated the quality control of endorsing scale with fit statistics to detect responding errors, which will be further immediately reconfirmed by technicians once patient ends the CAT assessment.

**Results:**

The results show that endorsed items could be shorter on CAT (*M *= 13.42) than on NAT (*M *= 23) at 41.64% efficiency in test length. However, averaged ability estimations reveal insignificant differences between CAT and NAT.

**Conclusion:**

This study found that mobile nursing services, placed at the bedsides of patients could, through the programmed VBA-Excel CAT module, reduce the burden to patients and save time, more so than the traditional NAT paper-and-pencil testing appraisals.

## Background

Many hospitals in Taiwan have implemented a mobile computer cart, which is also called a computer on wheels (COW) or a tablet PC. It is small, compact, wireless, and easy to fit a ward. Nurses or physicians can easily roll up a COW to access charts and perform their rounds. Besides, occupational therapists can help patients self-rate their ability to perform tasks in living and working environments with this promising device.

The Motion C5 [1], also known as the mobile clinical assistant (MCA), which integrates technology from Intel^® ^Health, not only brings reliable, automated, patient data management directly to the point of care, but also combines and increases productivity and improves overall quality of care. Although many studies [2,3] have addressed the fact that clinicians and medical staff prefer a tablet PC over a mobile cart with a laptop computer for supporting electronic clinical documentation, it is of interest to study whether computerized adaptive testing (CAT), based on item response theory (IRT) [4], could further enrich the advantage of using a tablet PC in evaluating patients' activities of daily living (ADL) functions.

There are many clinical functional scales, such as the Barthel Index, Frenchay Activities Index, Functional Independence Measure, Berg Balance Scale, Fugl-Meyer Motor Assessment Scale, Wolf Motor Function Test, Stroke Impact Scale and others. The psychometric properties of these scales are often investigated using classic test theory where a raw score is generally used to describe a patient's level of ADL function. CAT, based on IRT, is a method of administering tests that adapts to an examinee's latent trait level. CAT can save time and alleviate burden to patients and technicians than traditional non-adaptive paper-and-pencil or computerized-based assessments [5-7]. CAT has attracted many researchers' attention because it has a better control of item exposure and a less cost consumption on item development in medical and healthcare professions.

### Purposes

The aim of this study is to verify how CAT can save time and reduce burden for patients and technicians, through the following three steps: (a) A simulation study was conducted to justify that CAT needs a shorter test length than traditional non-adaptive testing(NAT) to achieve a similar degree of measurement precision; (b) A graphical CAT multimedia on a tablet PC was demonstrated to collect data of ADL clinical functional measures in stroke patients; (c) The quality control fit statistics and unexpected standardized residuals derived from Rasch analysis [8] was used to detect responding errors, which were further immediately reconfirmed by technicians with regard to patient's response.

## Methods

### 1. Activities of daily living

ADL is defined by the MedicineNet.com Medical Dictionary as "the thing we normally do in daily living including any daily activity we perform for self-care (such as feeding ourselves, bathing, dressing, grooming), work, homemaking, and leisure." The evaluation for ADL includes physical and mental skills. In the area of physical or occupational therapy, ADL reflects how well a disabled patient or individual recovering from a disease or an accident can function in daily life. It is also used to determine how well patients relate and participate in their environment.

### 2. Basic ADL versus Instrumental ADL

Basic ADL evaluated by the Barthel Index (BI) are those skills needed in typical daily self care. An evaluation would, in part, consist of bathing, dressing, feeding, and toileting. On the other hand, instrumental ADL refers to skills beyond basic self care that evaluates how individuals function within their homes, workplaces, and social environments. Instrumental ADL may include typical domestic tasks, such as driving, cleaning, cooking, and shopping, as well as other less physically demanding tasks, such as operating electronic appliances and handling budgets.

Hsueh et al. [9] stated that basic ADL does not capture significant losses in higher levels of physical function or activities that are necessary for independence in the home and community [10]. Several authors [5,11] recommend combining basic ADL and instrumental ADL to comprehensively measure ADL function and avoid a ceiling effect exhibited in the BI and a floor effect exhibited in the Frenchay Activities Index (FAI; measuring IADL) [5,11]. Such a combined scale is expected to be more responsive and have a wider range than either of the individual measurements [12,13].

### 3. The combination of BI & FAI

Hsueh and his colleagues [9] performed Rasch analysis to link the BI and FAI into a combined scale using the WINSTEPS software [14] (Linacre, 2007), which is one of the most widely used programs for Rasch measurement [15]. The partial credit model [16] in which each item has a unique rating scale structure [17] was fit to the data. The middle response category was collapsed due to disordered steps and simplicity. Two items were removed because of poor fit. The final version of the combined scale consisted of 23 dichotomous items.

### 4. Data source and generation

We used these 23 dichotomous items, shown in Table 1, to build an item bank to assess comprehensive ADL function in stroke patients. We programmed a VBA-Excel CAT module to (a) gather simulation data, (b) verify that CAT only needs a shorter test length to achieve the same measurement precision as non-adaptive testing, (c) illustrate how a tablet PC works graphically, and (d) report how the Rasch-specific quality control helps detect aberrant responses to ensure measurement quality.

**Table 1:** Combined 23 items of BI & FAI

No.	Items	Difficulty	SE
1	FAI13: household/car maintenance	4.73	0.31
2	FAI14: reading books	4.72	0.31
3	FAI15: gainful work	4.01	0.26
4	FAI12: gardening	3.75	0.25
5	FAI9: actively pursuing hobbies	3.53	0.24
6	FAI11: travel outings/car rides	3.52	0.24
7	FAI1: preparing main meals	3.24	0.23
8	FAI3: washing clothes	3.19	0.23
9	FAI2: washing up	3.09	0.22
10	FAI5: heavy housework	2.75	0.22
11	FAI4: light housework	1.95	0.21
12	FAI10: driving a car/bus travel	1.83	0.2
13	FAI6: local shopping	0.59	0.21
14	BI2: bathing	0.55	0.21
15	BI10: stairs	-0.72	0.22
16	BI4: dressing	-0.77	0.22
17	BI9: mobility	-2.85	0.26
18	BI7: toileting	-3.48	0.27
19	BI8: transfer	-3.99	0.27
20	BI3: grooming	-6.77	0.32
21	BI6: bladder control	-7.09	0.34
22	BI5: bowel control	-7.33	0.35
23	BI1: feeding	-8.41	0.44

	Mean	0	0.26
	SD	4.19	0.06

As in Hsueh et al. [9], we simulated 1,000 persons from a normal distribution with mean 1.17 and standard deviation 3.94. Treating the parameters in Table 1 as true values, we simulated a data set of 1,000 × 23 according to the Rasch dichotomous model. This whole data set, representing NAT, was treated as a base-line so that the performance of CAT can be assessed. It was expected that CAT, with a shorter test length, can achieve a similar degree of measurement precision as NAT.

### 5. IRT-based CAT

**Figure 1:**
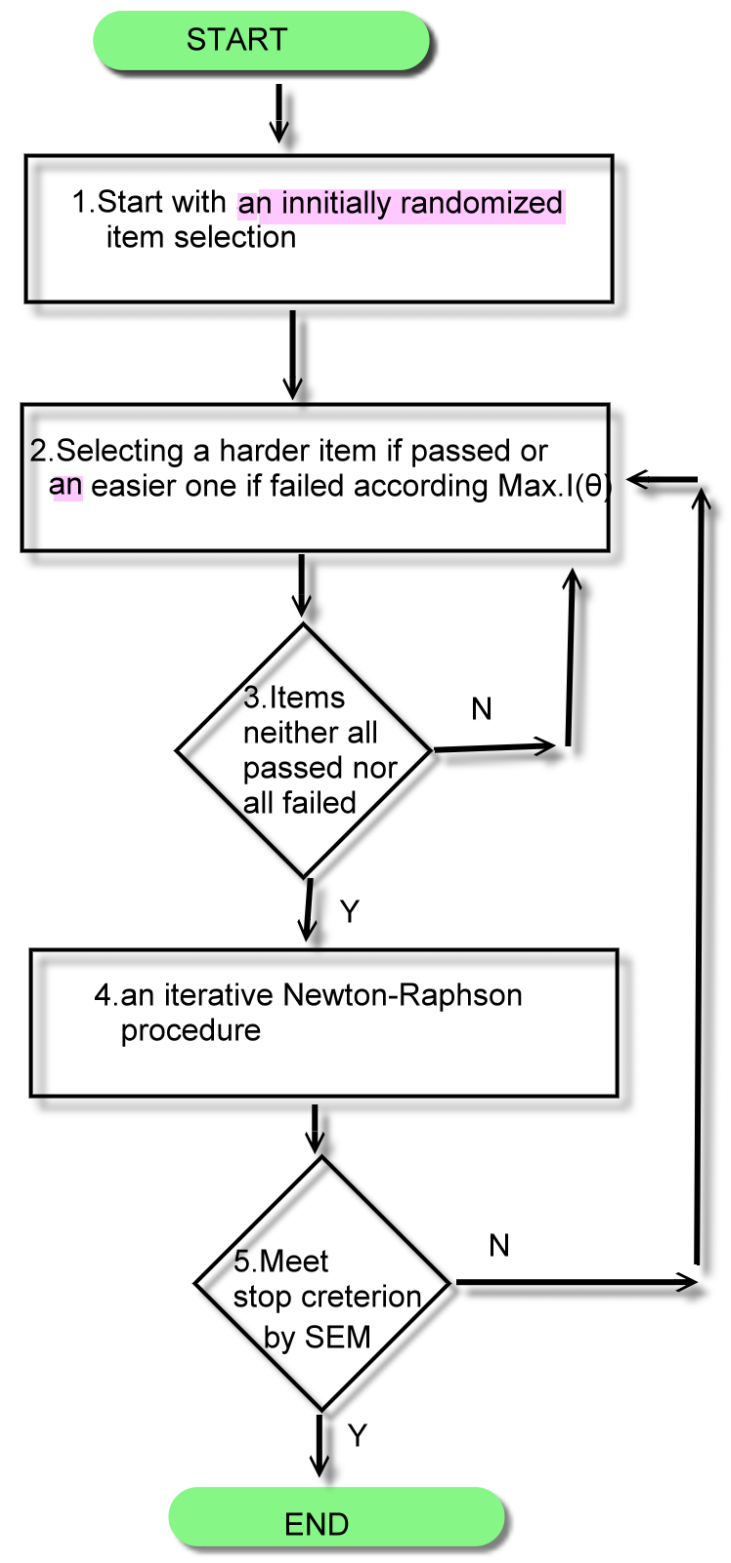
**Procedure and flowchart of CAT**.

We programmed a VBA module in Microsoft Excel in compliance with the flowchart in Figure 1. It has been found [9] that the person separation reliability (similar to Cronbach's alpha) was .94, and the persons followed a normal distribution with mean 1.17 and standard deviation 3.94. Under such a case, the mean standard error measurement across persons was 0.965, which served as the stopping rule of CAT.

#### There are three major concepts in CAT

##### (1). Individual measures estimated in CAT

The first step in CAT is to estimate individual person measure, which is often done by locating the maximum of the log-likelihood function for person measure using an iterative Newton-Raphson procedure [17]. This algorithm searches for the mode (rather than the mean) of each person's log-likelihood function through iteratively minimizing the ratio of first over second derivatives of the log-likelihood function. The provisional person measure is derived at individual iterations (or CAT steps) by the previous estimation minus its converged rate. Interested readers can refer to the textbook of Item Response Theory for Psychologists [17] or visit website at  for detailed CAT procedure.

##### (2) Person SE controlled in CAT to attain its desirable test reliability

The second step in CAT is to assure appropriate measurement precision. As stated above, the standard error of measurement (SEM) was set at 0.965, in order to achieve a test reliability of .94, as shown in the flowchart in Figure 1. SEM is a function of the summation of item information for those items that have been administered. The next item to be administered is the item in the item bank that provides the highest information about the person measure.

##### (3) Multimedia CAT along the patient bedside

The third step in CAT is the application in healthcare settings. The VBA-Excel based CAT module demonstrates how those unidimensional 23 items can assess comprehensive ADL function in stroke patients and how the unexpected response with a Z-score beyond ± 2 [18] could be examined as patient made questionable responses, to which needs highly alert or even to redo them for guaranteeing the quality of endorsement.

## Results

### Efficiency of CAT

Among the 1,000 simulated persons, 826 had neither a zero nor a perfect raw score. As shown in Table 2, CAT did not yield person measure estimates that were statistically different from non-adaptive testing (*p *= .78); and CAT had a shorter test length than NAT (*p *< .01). NAT took all the 23 items, whereas CAT took an average test length of 13.42 items. Thus, the efficiency of CAT was supported. Each round of a CAT test can save at least five minutes to both patient and occupational therapist, and can reach a much more accurate set of responses through outline Z-score examination than NAT.

**Table 2:** Comparison of CAT and non-adaptive testing (NAT) in measurement efficiency with the t-test

	Mean	Variance	Observed	Maximum	Minimum	*p-value*
Estimated Ability:						
NAT	-0.23	9.19	826	5.22	-8.62	.78
CAT	-0.20	8.92	826	4.76	-8.62	

Test length:						
NAT	23	0	826	23	23	< .001
CAT	13.42	62.04	826	23	5	

### CAT on a tablet PC in healthcare settings

#### (1) Provisional person measure

To demonstrate ADL assessment by CAT on a tablet PC, we show in Table 3 the item selection procedures and the stopping rules. At the beginning, a randomly selected item (e.g., FAI6 with difficulty 0.59) was administered to a person whose provisional person measure was set at zero. The simulated response was "fail" (scored as 0) and an easier item (i.e., BI4 with difficulty -0.77) was administered. The simulated response was "passed" (scored as 1). Since there were both "pass" and "fail", the person measure can be updated through the Newton-Raphson iteration approach [17], which was 0.21, with SEM 2.43 at step 2.

**Table 3:** A case of the CAT responding process (Yes = 1, No = 0) with a sequence of item selection.

Step	Response	Ability	SEM	Items	Difficulty
1	0	-	2.77	FAI6: local shopping	0.59
2	1	0.21	2.43	BI4: dressing	-0.77
3	1	0.87	1.86	BI2: bathing	0.55
4	1	1.76	1.39	FAI10: driving a car/bus travel	1.83
5	1	2.42	1.15	FAI4: light housework	1.95
6	1	3.07	1.00	FAI5: heavy housework	2.75
7	0	2.65	0.96	FAI2: washing up	3.09

#### (2) Item selection and stop criterion

Given a provisional person measure of 0.21, the next item to be administered was the one that provided the highest information about the person, which was BI2 with 0.55. The simulated response was "passed" and the person measure was updated as 0.87 with SEM 1.86 at step 3. This procedure repeated until step 7 where FAI2 with difficulty 3.09 was administered and the updated person measure was 2.65 with SEM 0.96. CAT stopped because SEM was smaller than the criterion of 0.965.

#### (3) Aberrant responses examined by Z-score

##### 1. Graphical multimedia CAT along the patient bedside

**Figure 2:**
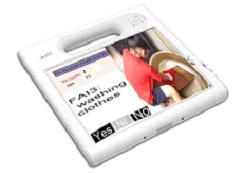
**CAT implemented on a tablet PC**.

**Figure 3:**
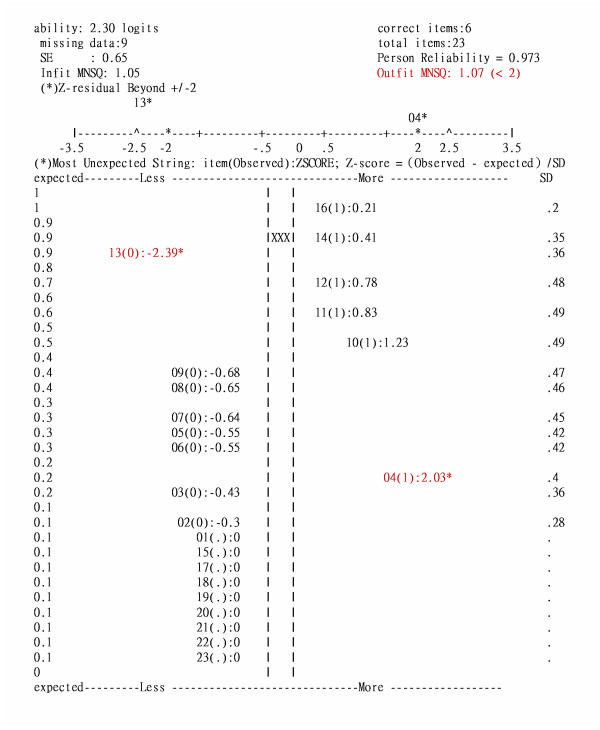
**Z-scores scatter diagram for the items to which the examinee responded**. Note. *p < .05; item number(observed score): Z-score. XXX: person estimation equal to ln(P/(1-P) = ln(.9/.1) = ln(9) = 2.3 logits.

The screenshot of the CAT implementation on a tablet PC is shown in Figure 2. Linacre [19] stated that a person would be deemed a severe aberrant responder to the test when the outfit mean square error (MNSQ) is greater than 2.0 together with a *Z *beyond ± 2 (the expected outfit MNSQ is 1 for a good fit [20]). An occupational therapist can use this statistic to check whether the response pattern is aberrant. If not so like the illustrator of Outfit MNSQ 1.07 on the upper-right corner in Figure 3, one then has confidence that the responses can reveal valuable information about the respondent

##### 2. Outline aberrant responses examined by Z-score

In Figure 3, one can easily observe that the person with ability 2.30 failed on item 13 with difficulty 0.59, as shown in the upper-left of Figure 3. This was an aberrant response because the probability of success for such a person on such an item was as high as .90. Another aberrant response was found on the lower-right of Figure 3, where the person passed item 4 unexpectedly because the probability of success on that item was as low as .20.

Figure 3 can be plotted on the screen of the tablet PC once the patient completes the CAT. The patient in this case might be required to complete these two tasks again in order to submit accurate responses to the healthcare database.

## Discussion

The item bank was chosen from Hsueh et al. [9] which measures ADL for stoke patients. We successfully developed a VBA-Excel CAT module and demonstrated how CAT can be used to reduce patient and proxy burdens, and improve data collection and quality measurement. Through simulations, it was found that CAT can save up to 42% of test length and achieve a very similar degree of measurement precision as NAT. This is consistent with the literature [5-7]. This study also found that mobile nursing services along the bedsides of patients, through the programmed VBA-Excel graphical CAT module, is much less burdensome to patients and time saving than traditional NAT appraisals.

IRT-based CAT algorithms have been developed in educational testing for several decades and much is known about their functioning in comparison to NAT [6,22,23]. CAT utilizes the invariance property under the Rasch or IRT models to create an algorithm by which each person receives a test that is tailored to the person's level so that the questions are neither too difficult nor too easy and usually contain fewer items than conventional non-adaptive measures [19].

## Conclusion

Mobile nursing services through the programmed VBA-Excel CAT module can reduce the burden to patients and proxies and save time, more so than the traditional non-adaptive assessing appraisals. With the networking and rapidly growing mobile point of care development in hospitals, IRT-based assessing appraisal is more in line with real-world test, especially used in healthcare. We expect that over the years this mobile framework of graphical CAT assessing patient ADL as piloted in this study will draw more research attention.

## List of abbreviations

CAT: computerized adaptive testing; IRT: all answered items; CTT: classic test theory; AAL: all answered items; VBA: visual basic for application; ADL: activities of daily living; COW: computer on wheels; MCA: mobile clinical assistant; MNSQ: mean square errors; ZSTD: Z-standardized; SEM: standard error measurement.

## Competing interests

The authors declare that they have no competing interests.

## Authors' contributions

TC collected all data and built up the database, designed and performed the statistical analysis and wrote the manuscript. HW and WW contributed to the development of the study design and advised about the performance of the statistical analysis. WW contributed to the revision of manuscript. The analysis and results were discussed with the five authors together. HW, WW, RC and WC revised the manuscript critically several times. WW advised the CAT programming, helped with interpreting the results and helped to draft the manuscript. All authors read and approved the final manuscript.
